# Sodium nitrate regulates senescence accompanied by aortic atherosclerosis in ApoE^−/−^ mice through the miR-34a/FGF-21 axis

**DOI:** 10.3389/fphar.2025.1562321

**Published:** 2025-03-05

**Authors:** Ning Tao, Zhichao He, Han Duan, Liang Wang, Jing Yi, Jingyuan Shao, Lin Lv, Junzhao Duan, Hu Cao, Xiwen Dong, Hua Wang

**Affiliations:** ^1^ College of Life Science, Anhui Medical University, Hefei, China; ^2^ Academy of Military Medical Sciences, Beijing Institute of Radiation Medicine, Beijing, China; ^3^ Department of Dermatology, Air Force Medical Center, PLA, Beijing, China

**Keywords:** atherosclerosis, senescence, sodium nitrate, miR-34a, FGF21

## Abstract

**Introduction:**

Increasing evidence indicates that cellular senescence is a significant risk factor for atherosclerosis (AS).

**Methods:**

In the present study, we used an apolipoprotein E knockout (ApoE^−/−^) mouse model to address the effect of sodium nitrate on senescence accompanied by atherosclerosis. After sodium nitrate intervention, the degree of AS pathological and cellular senescence changes was evaluated in mouse aortic. At the same time, an H_2_O_2_-induced human arterial endothelial cell (HAoEC) senescence model was established to verify the role of miR-34a in AS-associated senescence.

**Results:**

We observed that sodium nitrate decreased the Oil Red O-positive area, reduced the serum cholesterol (CHO) and triglyceride (TG) concentrations, and relieved inflammatory reactions in ApoE^−/−^ mice. Moreover, the SA-β-Gal-positive area, the expression of cell cycle regulation-related genes and miR-34a in the aorta decreased after sodium nitrate treatment. Furthermore, sodium nitrate upregulated the expression of FGF21 by inhibiting the expression of miR-34a, thereby rescuing the senescent phenotype of HAoECs. These results suggested that sodium nitrate could rescue the endothelial cell senescence phenotype and alleviate aortic atherosclerosis in ApoE^−/−^ mice by regulating the miR-34a/FGF21 axis.

**Discussion:**

These findings might lead to the introduction of a new therapy for senescence-related diseases in the future.

## Graphical Abstract

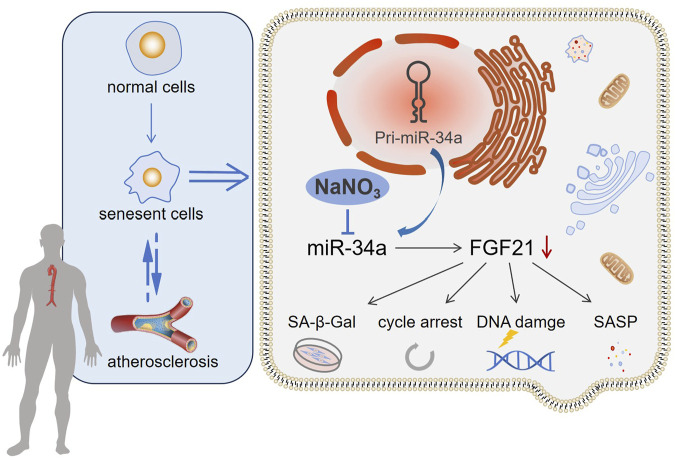

## 1 Introduction

Atherosclerosis (AS) is a chronic progressive pathological process that refers to the continuous deposition and aggregation of lipids in the intima of arteries and some other components in the blood; this process leads to damage of the intima and proliferation and movement of smooth muscle cells and collagen fibers to the intima and is accompanied by various degrees of vascular pathological processes, such as necrosis and calcification (Libby et al., 2019). Inflammation theory has been the most recognized theory in recent years. AS is not a simple lipid deposition disease. It is considered a long-term, chronic, low-level inflammatory response process (Tedgui and Mallat, 2006). Inflammation plays a vital role in the occurrence and development of AS and is a marker and important driver factor of pathological vascular remodeling in AS.

Senescence, a kind of stress-induced and persistent cell cycle arrest, is induced by many stimulating factors, including carcinogenic signal transduction, telomere dysfunction, DNA damage, mitochondrial dysfunction, and oxidative stress (He and Sharpless, 2017; Lopez-Otin et al., 2023). During the senescence process, cells secrete various characteristic factors, collectively known as the senescence-related secretory phenotype (SASP), such as chemokines, interleukins, and proteases (Borghesan et al., 2020; De Cecco et al., 2019). These factors promote the senescence of local cells and may even cause aging of the system via a paracrine pathway (Tasdemir and Lowe, 2013). Increasing evidence indicates that cellular senescence and aging are closely related to the occurrence and development of various age-related diseases (Wiley and Campisi, 2021).

Many studies have verified that AS is closely related to premature aging in organisms, and AS plaques also exhibit phenotypes related to cell senescence, such as decreased cell proliferation ability, growth arrest and apoptosis, DNA damage, and mitochondrial dysfunction (Xu et al., 2020). Simultaneously, various inflammatory factors are secreted to promote the development of AS and inhibit plaque repair. In addition, a variety of metalloproteinases are secreted by senescent endothelial cells, monocytes, macrophages, and foam cells and can promote the thinning of fiber caps and cause instability of atherosclerotic plaque (Wang et al., 2015). This means that senescent cells may be an important source of local, chronic, and low-level inflammatory and plaque instability factors while providing various additional plaque instability factors (Stojanovic et al., 2020; Childs et al., 2018). Senescence may play an important role in the entire process, from the occurrence of AS to plaque rupture. Different types of senescent cells play important roles in the process of atherosclerosis (Childs et al., 2016). The senescence of endothelial cells, vascular smooth muscle cells, macrophages, and other cells can be observed at the early stage of AS. These senescent cells can further expand the necrotic core, accelerate the degeneration of the extracellular matrix, decrease the thickness of the fiber cap, erode it, calcify it, and assist angiogenesis in plaques (Bentzon et al., 2014).

The beneficial effects of inorganic nitrates on various biological processes have been revealed in recent decades. Dietary nitrate can act as a reservoir of NO in the body through the nitrate-nitrite-NO pathway. Nitrate has a variety of biological activities similar to NO, such as improving exercise ability, protecting the digestive system, lowering blood pressure, and assisting in tumor treatment (Qin and Wang, 2022).

Bakker et al. reported that dietary nitrate can play an anti-inflammatory role by regulating the expression level of inflammatory factors (Bakker et al., 2016). Clinical data have shown that long-term nitrate supplementation can improve the degree of arteriosclerosis and reduce systolic blood pressure and proinflammatory factor levels in elderly volunteers (Rammos et al., 2014). Daily supplementation with nitrate can alleviate cellular senescence induced by D-galactose and natural senescence in mice and inhibit the occurrence of age-related lesions in the liver (Wang et al., 2018). However, the effect of dietary nitrate on senescence accompanied by AS and the underlying mechanism is still unknown.

In this study, ApoE^−/−^ mice fed a high-fat diet were used to establish a mouse model of aortic AS, and the therapeutic effect of sodium nitrate on AS and accompanying cell senescence was evaluated. In addition, we established an H_2_O_2_-induced senescence model in HAoECs to clarify the mechanism through which sodium nitrate alleviates cell senescence.

## 2 Materials and methods

### 2.1 Cell culture

Human aortic endothelial cells (HAoECs) were purchased from ATCC (United States). HAoECs were cultured in Dulbecco’s modified Eagle’s medium (DMEM, 11965092, Gibco, United States) supplemented with 10% fetal bovine serum (FBS, 12003C, Sigma, United States).

### 2.2 Cell senescence model and treatment

HAoECs were treated with H_2_O_2_ (600 μM, 7722-84-1, Sinopharm Chemical Reagent, China) for 24 h to establish a cell senescence model and then cocultured with 50 μM sodium nitrate (221341, Sigma, United States). To verify the specific effects of miR-34a, HAoECs were transfected with 10 nM miR-34a mimic, inhibitor or corresponding NC (Sangon Biotech, China) according to the manufacturer’s instructions for the RNATransMate reagent (E607402, Sangon Biotech, China). All four miRNA were dissolved in DEPC water provided with the kit. FGF21 recombinant protein (50 ng/mL) (rhFGF21, HY-P7345, MedChemExpress, United States) were also used to treat HAoECs concomitantly with miR-34a mimic. The sequences of the miR-34a mimic, inhibitor and the corresponding control sequences are listed in Table 1.

**TABLE 1:** The sequences of the miR-34a mimic and miR-34a inhibitor.

Name	Sequence
miR-34a mimic NC	Sense: UUGUACUACACAAAAGUACUG Antisense: GUACUUUUGUGUAGUACAAUU
miR-34a mimic	Sense: UGGCAGUGUCUUAGCUGGUUGU Antisense: AACCAGCUAAGACACUGCCAUU
miR-34a inhibitor NC	CAG​UAC​UUU​UGU​GUA​GUA​CAA
miR-34a inhibitor	ACA​ACC​AGC​UAA​GAC​ACU​GCC​A

### 2.3 Animal studies

ApoE^−/−^ mice (8 weeks old, male, with a C57BL/6 background) were obtained from Beijing Vital River Laboratory Animal Technology Co., Ltd. (China). All animals were maintained on a 12 h/12 h light/dark cycle, with a temperature of 22°C ± 2°C and controlled humidity ranging from 50% to 60%. 36 ApoE^−/−^ mice were randomly divided into 3 groups (12 mice per group: three aorta of mice were used for oil red o-staining, three for SA-β-Gal staining, three for immunohistochemical detection, and the rest for RNA extraction): normal group (NFD), fed normal chow (1022, Beijing HFK Bioscience CO., LTD, China) and double distilled water for 12 weeks; high-fat group (HFD), fed high-fat chow (H10540, Beijing HFK Bioscience CO., LTD, China) containing 40 kcal % fat (1.254% cholesterol by weight), 20% kcal protein and 40 kcal % carbohydrates for 12 weeks; and sodium nitrate treated group (HFD + NaNO_3_), fed high-fat chow and drank 1 g/mL sodium nitrate for 12 weeks. All animal experiments were performed under protocols approved by the Institutional Animal Care and Use Committee of the Laboratory Animal Center (IACUC DWZX-2021-714).

### 2.4 Serum lipid profile

Whole blood was collected from the canthal vein of mice at weeks 0, 3, 6, 9, and 12, and left at room temperature for 30 min, then at 4°C for 30 min, and finally centrifuged at 3,000 rpm for 10 min at 4°C to isolate serum. Total cholesterol (CHO) (S03042, Leidu, China) and triglyceride (TG) were measured using a total cholesterol determination kit and triglyceride determination kit (S03027, Leidu, China), respectively.

### 2.5 ELISA

Blood was collected from the angular vein of ApoE^−/−^ mice at weeks 3, 6, 9, and 12, and serum was sampled. The expression of IL-6 and TNF-α was detected by using an ELISA kit (MEK1016, Boster, United States) according to the manufacturer’s protocol. Briefly, the precoated 96-well plate was equilibrated to room temperature and incubated with 50 μL of diluted standard and mouse serum. The cells were washed three times with 1× wash buffer for 1 min each. Then, 50 μL of biotin-labeled antibody was added to the 96-well plate and incubated for 60 min. After washing with wash buffer, 50 μL of streptavidin-labeled horseradish peroxidase was added, and the mixture was incubated for 15 min. Then, the cells were washed again, and 50 μL of mixed substrate A and substrate B were added to each well (Substrate A: Substrate B = 1:1). All of the above operations were performed at room temperature. Finally, the analysis was performed using a Q-View Imager LS (QuansysBio, United States).

### 2.6 Oil red O staining

To quantify the burden of atherosclerotic plaques, the adventitial fat was removed from the harvested aortas before fixation. After fixation in a neutral formalin solution overnight, the aortas were washed with PBS and then incubated in Oil Red O solution (G1015, Servicebio, China) at RT for 1 h. After the PBS washes, the samples were imaged via optical microscopy (Nikon, Japan), and the percentage of positive staining was analyzed via ImageJ software (NIH, United States).

### 2.7 SA-β-Gal staining

At the endpoint of the experiment, the mice were sacrificed under excessive anesthesia with pentobarbital sodium. After the aortas were isolated, SA-β-Gal staining was performed following the manufacturer’s instructions (C0602, Beyotime, China), and the results were visualized via optical microscopy (Nikon, Japan). The percentage of positive staining was calculated using ImageJ software (NIH, United States).

### 2.8 Immunohistochemistry

At the endpoint of the experiment, the aortas of the ApoE^−/−^ mice were removed after cardiac perfusion with sterile saline, and the surface adipose tissue was removed in PBS. The incised aortas were fixed in 4% paraformaldehyde solution and embedded in paraffin before sectioning. After antigen retrieval, the sections were placed in a 3% hydrogen peroxide solution that blocks endogenous peroxidases. The paraffin sections were blocked with a 3% BSA solution (GC305010, Servicebio, China) for 30 min at RT and incubated with anti-P16 (dilution ratio: 1:600; ab241543, abcam, UK) overnight at 4°C. The next day, the sections were incubated with goat anti-rabbit IgG-HRP (dilution ratio: 1:200; GB23303, Servicebio, China) for 50 min at RT. Then, a DAB chromogenic solution (G1212, Servicebio, China) was added dropwise for chromogenic development, and a hematoxylin staining solution (G1004, Servicebio, China) was used for counterstaining. After the tissue was covered with mounting gel (G1404, Servicebio, China), it was photographed under a light microscope (Nikon, Japan) for observation. The P16-positive area ratio (positive area ratio = positive area/tissue area) was calculated with Aipathwell software (Servicebio, China), and the P16 expression level was evaluated.

### 2.9 Intracellular SA-β-Gal detection

HAoECs were cultured in 12-well plates at a density of 100,000 cells/well and treated with the corresponding reagents described in Section 2.2 for 24 h. Then, the culture medium was discarded, the cells were fixed with 0.5 mL of staining fixation solution for 15 min, and the cells were subsequently incubated with 0.5 mL of SA-β-Gal staining working fluid overnight. Five visual fields were observed through an optical microscope, and the percentage of positively stained areas was calculated.

### 2.10 Cell proliferation assay

HAoECs were seeded in 96-well plates at a density of 8,000 cells/well. After cell adhesion, the corresponding reagents described in Section 2.2 was added, and the cells were cultured for 96 h. After the cells were incubated with CCK-8 working solution (C0602, Beyotime, China) at 37°C for 1.5 h, the optical density (OD) at a wavelength of 450 nm was measured with a microplate reader (Thermo, United States).

### 2.11 Real-time PCR analysis

An RNA Rapid Extraction Kit (RN001, ES Science, China) was used to extract RNA from mouse aortas or HAoECs according to the manufacturer’s protocol. A Rapid Reverse Transcription Kit (RT001, ES Science, China) was used to synthesize complementary DNA, and PCR was performed on an ABI 7500 Fast (Applied Biosystems, United States) using qPCR SYBR Green Master Mix (11184ES03, Yeasen, China). All the results were calculated using the 2^−ΔΔCT^ method by normalization to the expression of GAPDH (human), Actin (mouse) or U6. The sequences of primers used are listed in Table 2 (the primers for the internal reference gene U6 was obtained from the reagent kit).

**TABLE 2:** The sequences of the real-time PCR primers.

Primer name^a^	Forward primer sequence	Reverse primer sequence
Actin-Mus	TCTTTGCAGCTCCTTCGT	GACCCATTCCCACCATC
P53-Mus	AACGCTTCGAGATGTTCC	GTTTGGGCTTTCCTCCTT
P21-Mus	GATCCCCTTTGCCACTC	TCA​CCA​GAT​TAA​CCC​TCC​A
P16-Mus	CGTGCGATATTTGCGTTC	ACGTTCCCAGCGGTACA
RB-Mus	CAA​AAG​AAG​TGC​TGA​AGG​C	CCGCTGGGAGATGTTTAC
IL-1β-Mus	TGT​CCT​GAT​GAG​AGC​ATC​C	AAGGTCCACGGGAAAGAC
IL-6-Mus	AGC​CCA​CCA​AGA​ACG​ATA​G	GGT​TGT​CAC​CAG​CAT​CAG​T
IL-8-Mus	CAC​TCC​ACT​ATG​GGC​TGT​T	TGGGGCACTGAAGACAA
TNF-α-Mus	CGCTGAGGTCAATCTGC	GGCTGGGTAGAGAATGGA
FGF21-Mus	GGTCAAGTCCGGCAGAG	CGCCTACCACTGTTCCAT
GAPDH-Hu	CCTTCCGTGTCCCCACT	GCCTGCTTCACCACCTTC
P53-Hu	GAG​GTT​GGC​TCT​GAC​TGT​ACC	TCC​GTC​CCA​GTA​GAT​TAC​CAC
P21-Hu	ATG​A-GTT​GGG​AGG​AGG​CA	CTGAGCGAGGCACAAGG
P16-Hu	GAT​CCA​GGT​GGG​TAG​AAG​GTC	CCC​CTG​CAA​ACT​TCG​TCC​T
RB-Hu	TAA​GAA​TGG​CCC​TAG​AGT​GG	TGC​TAC​AAA​AGA​AGG​CAA​AGT
IL-1β-Hu	TGT​CCT​GAT​GAG​AGC​ATC​C	AAGGTCCACGGGAAAGAC
IL-6-Hu	TGAGGAGACTTGCCTGGT	GGGTCAGGGGTGGTTATT
IL-8-Hu	CAG​TGA​AGA​TGC​CAG​TGA​AA	CAA​CCC​TAC​AAC​AGA​CCC​A
TNF-α-Hu	GAG​GCC​AAG​CCC​TGG​TAT​G	CGG​GCC​GAT​TGA​TCT​CAG​C
FGF21-Hu	TTTCTGTGCTGGCTGGTC	GCTGGGCATCATCTGTGT
miR-34a-5p	TGG​CAG​TGT​CTT​AGC​TGG​TTG	Provided by the kit

^a^ Full name of gene: Actin (actin, beta), P53 (transformation related protein 53), P21 (cyclin dependent kinase inhibitor 1A), P16 (cyclin dependent kinase inhibitor 2A), RB (RB transcriptional corepressor 1), IL-1β (interleukin 1 beta), IL-6 (interleukin 6), IL-8 (C-X-C motif chemokine ligand 15), TNF-α (tumor necrosis factor), FGF21 (fibroblast growth factor 21), GAPDH (glyceraldehyde-3-phosphate dehydrogenase), miR-34a-5p (microRNA 34a).

### 2.12 Western blot

HAoECs were collected and lysed in RIPA buffer (P0013C, Beyotime, China) supplemented with protease inhibitors, after which the protein concentration was detected using a BCA protein quantitative kit (23225, Thermo Scientific, United States). The proteins were separated by 12% SDS‒PAGE and transferred to a polyvinylidene fluoride (PVDF) membrane. Then, the PVDF membrane was blocked with 5% skim milk powder and incubated overnight with the corresponding primary antibodies at 4 °C: anti-RB (dilution ratio: 1:500, #9309; Cell Signaling Technology, United States), anti-P53 (dilution ratio: 1:1000, #48818; Cell Signaling Technology, United States), anti-P21 (dilution ratio: 1:1000, #80772; Cell Signaling Technology, United States), and anti-GAPDH (dilution ratio: 1:5000, #10494-1-AP; Proteintech, China). The next day, the membranes were washed with TBST four times for 5 min each time and incubated with the following secondary antibodies for 1 h at RT: goat anti-rabbit IgG-HRP (dilution ratio: 1:3000, #GB23303; Servicebio, China) and goat anti-mouse IgG-HRP (dilution ratio: 1:000, #GB23301; Servicebio, China). After washing with TBST four times, the signal was detected by using an enhanced chemiluminescence (ECL) kit (PE0010, Solarbio, China).

### 2.13 Immunofluorescence

The DNA damage foci of HAoECs were detected using a DNA Damage Assay Kit (C2035S, Beyotime, China) following the manufacturer’s instructions. Briefly, the treated cells were fixed with 4% paraformaldehyde at RT and coincubated with the rabbit monoclonal antibody γ-H2AX overnight at 4°C. Then, an anti-rabbit secondary antibody was added for 1 hour at RT in the dark. After 10 min of coincubation with DAPI to stain the nuclei, images were acquired using fluorescence confocal microscopy (CrestOptics, Italy). ImageJ software (National Institutes of Health, United States) was used to count the number of DNA damage foci (National Institutes of Health, United States).

### 2.14 Statistical analysis

Statistical analysis was performed using GraphPad Prism 7 (San Diego, United States). One-way analysis of variance (ANOVA) was used to compare multiple groups, and a t-test was used to compare two groups. The data are expressed as the mean ± SD, and a P value less than 0.05 was used to indicate statistical significance.

## 3 Results

### 3.1 Sodium nitrate treatment alleviated aortic atherosclerosis in ApoE^−/−^ mice

**FIGURE 1:**
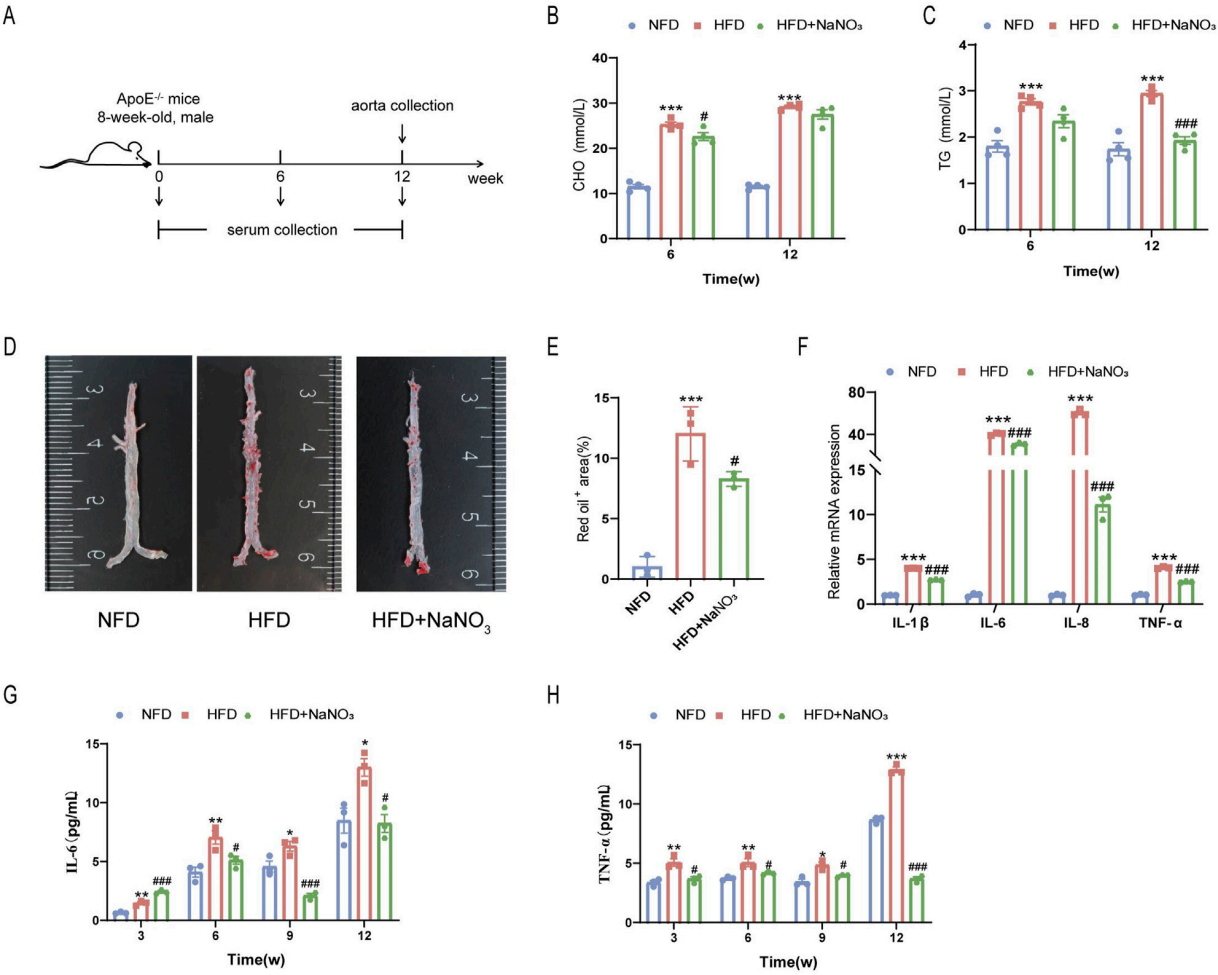
The effect of sodium nitrate on the AS. **(A)** Mice were treated as shown in the scheme. The concentrations of CHO **(B)** and TG **(C)** in the serum of the mice were detected. n = 4. **(D)** Representative image of Oil Red O staining of the aorta. **(E)** Statistical analysis of the data in **(D)**. n = 3. **(F)** QPCR was used to detect the expression levels of inflammatory factors in the aortas of the mice. n = 3. **(F)** The expression levels of the serum inflammatory factors IL-6 **(G)** and TNF-α **(H)** were detected. n = 3. *p < 0.05, **p < 0.01, ***p < 0.001, vs. the NFD group; ^#^p < 0.05, ^###^p < 0.001, vs. HFD group.

An AS mouse model was established through the consumption of a high-fat diet for 12 weeks to determine the effect of sodium nitrate on senescence accompanied by AS (Figure 1A). Mouse serum was collected at weeks 0, 3, 6, 9, and 12, and the mouse aorta was removed at the experimental endpoint. Our results showed that after the consumption of a high-fat diet, the concentrations of serum CHO (Figure 1B) and TG (Figure 1C) increased significantly, and sodium nitrate treatment decreased them. However, compared to the HFD group, the NaNO_3_ group had no statistically significant difference in TG concentration at 6 weeks and CHO concentration at 12 weeks. The Oil Red O staining area is a key indicator for evaluating the size of AS plaques, and our results showed that, compared with that in the HFD group, the area stained with Oil Red O decreased by approximately 30% in the sodium nitrate treatment group (Figures 1D, E). To evaluate the effect of sodium nitrate on atherosclerosis, we detected inflammatory cytokine expression in mouse aortas by using QPCR and found that the expression levels of IL-1β, IL-6, IL-8, and TNF-α, especially IL-8, were significantly decreased in the sodium nitrate treatment group (Figure 1F). We also detected the expression levels of the serum inflammatory factors IL-6 and TNF-α by using ELISA. The results showed that sodium nitrate treatment reduced the concentrations of serum IL-6 (Figure 1G) and TNF-α (Figure 1H). These results indicated that sodium nitrate could effectively alleviate atherosclerosis and reduce systemic and local inflammatory reactions.

### 3.2 Sodium nitrate treatment alleviated cellular senescence in ApoE^−/−^ mice

**FIGURE 2:**
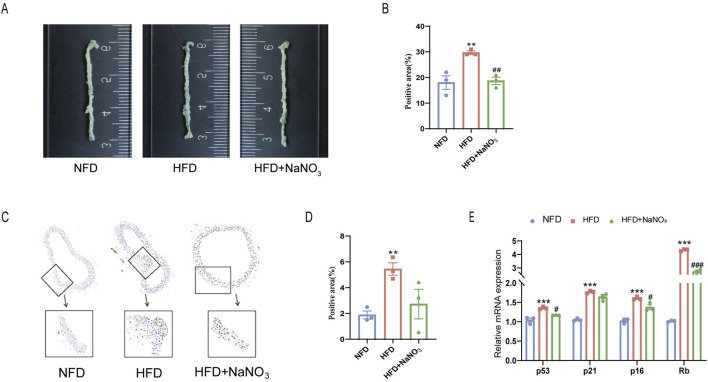
Effect of sodium nitrate on the senescence of aortic cells in ApoE^−/−^ mice. **(A)** Representative image of SA-β-Gal staining of the aorta. **(B)** Statistical analysis of the staining-positive area in **(A)**. n = 3. **(C)** Representative images of P16 expression in the aorta detected by immunohistochemistry. **(D)** Statistical analysis of the stained area in **(C)**. n = 3. **(E)** The expression levels of p53, p21, p16 and Rb. n = 3. **p < 0.01, ***p < 0.001, vs. the NFD group; ^#^p < 0.05, ^##^p < 0.01, ^###^p < 0.001, vs. HFD group.

To investigate the effect of sodium nitrate on the senescence of ApoE^−/−^ mice, we collected aortas from the mice for SA-β-Gal staining (Figures 2A, B). The results showed that a high-fat diet significantly increased the percentage of SA-β-Gal-positive cells, while sodium nitrate treatment significantly decreased the percentage of SA-β-Gal-positive cells. The immunohistochemistry results showed that the expression level of P16, a classic indicator of cellular senescence, was significantly lower in the sodium nitrate treatment group than in the HFD group (Figures 2C, D). Compared to those in the NFD group, the expression levels of p53, p21, p16, and Rb in the aorta were upregulated in the HFD group. Sodium nitrate treatment downregulated the expression of these senescence-related genes (Figure 2E). These results showed that sodium nitrate could alleviate cell senescence accompanied by aortic atherosclerosis in ApoE^−/−^ mice.

### 3.3 Sodium nitrate treatment alleviated the senescence of HAoECs

**FIGURE 3:**
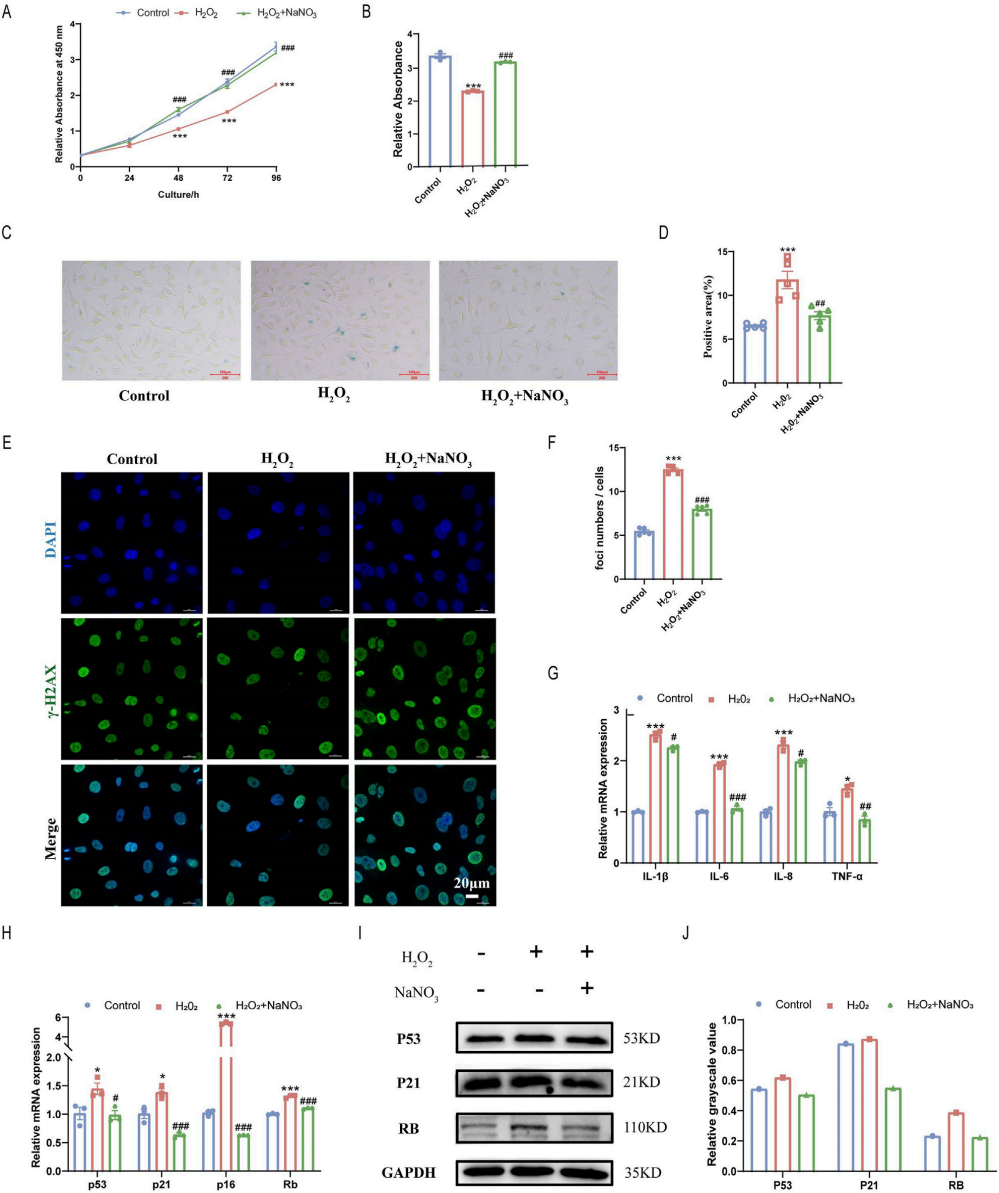
Effect of sodium nitrate on H_2_O_2_-induced senescence in HAoECs. **(A)** The CCK-8 method was used to evaluate the effect of sodium nitrate on the proliferation of HAoECs. **(B)** Statistical analysis of cell proliferation at 96 h **(A)**. n = 3. **(C)** Representative images of SA-β-Gal-stained HAoECs. Bar = 100 μm. **(D)** Statistical analysis of the stained area in **(C)**. n = 5. **(E)** Representative images of γ-H2AX staining of HAoECs. Bar = 10 μm. **(F)** Statistical analysis of the DNA damage foci in **(E)**. n = 5. **(G)** QPCR analysis of the mRNA expression of the inflammatory factors IL-1β, IL-6, IL-8, and TNF-α in HAoECs. n = 3. **(H)** QPCR was used to detect the relative expression levels of the cell cycle-related genes p53, p21, p16 and Rb in HAoECs. n = 3. **(I)** Western blotting was used to detect the expression of P53, P21, and RB in HAoECs. n = 3 **(J)** Quantitative analysis of the results in **(I)**. *p < 0.05, ***p < 0.001, vs. the control group; ^#^p < 0.05, ^##^p < 0.01, ^###^p < 0.001, vs. H_2_O_2_ group.

To further verify the effect of sodium nitrate on cell senescence, 600 μM H_2_O_2_ was used to induce senescence in the HAoECs. We examined the cell proliferation ability of HAoECs induced by H_2_O_2_ using a CCK-8 kit. H_2_O_2_ significantly inhibited the proliferation of HAoECs, and sodium nitrate treatment increased this ability by approximately 40% at 96 h (Figures 3A, B). The SA-β-Gal staining results also showed that sodium nitrate could reduce the increase in the percentage of H_2_O_2_-treated HAoECs (Figures 3C, D). The number of DNA damage foci induced by H_2_O_2_ was significantly reduced after sodium nitrate treatment (Figures 3E, F). Sodium nitrate treatment also downregulated the expression of the inflammatory cytokines IL-1β, IL-6, IL-8, and TNF-a (Figure 3G). Moreover, compared to those in the H2O2 group, the expression levels of cell cycle-related genes at the mRNA (p53, p21, p16, and Rb) levels were lower in the sodium nitrate treatment group (Figure 3H). But no statistical differences in the expression of the cell cycle-related protein between groups (Figures 3I, J).

### 3.4 Sodium nitrate could regulate the expression of miR-34a and its downstream gene FGF21

**FIGURE 4:**
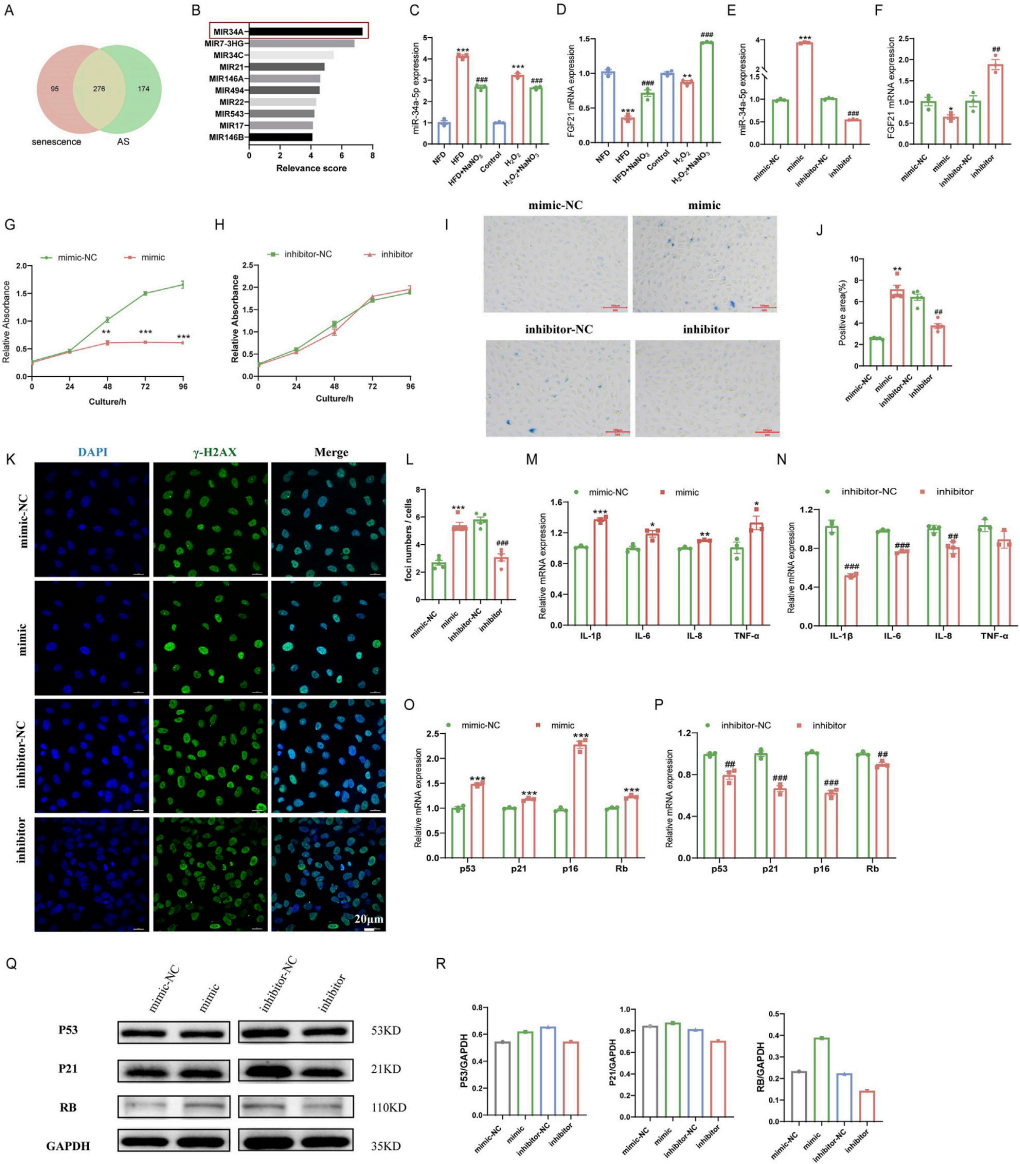
Effects of miR-34a and FGF21 in ApoE^−/−^ mice and HAoECs. **(A)** Venn plots for GeneCards database search for senescence and AS-related miRNA. **(B)** Top 10 miRNAs with the highest correlation scores associated with senescence in the common 276 miRNAs. **(C)** QPCR was used to detect the expression level of miR-34a in ApoE^−/−^ mice at 12 weeks and HAoECs at 24 h. n = 3. **(D)** QPCR analysis of FGF21 mRNA expression in ApoE^−/−^ mice at 12 weeks and HAoECs at 24 h. n = 3. **(E)** QPCR was used to detect the expression levels of miR-34a in HAoECs after treatment with the miR-34a mimic or inhibitor at 24 h. n = 3. **(F)** QPCR analysis of FGF21 mRNA expression in HAoECs after treatment with the miR-34a mimic or inhibitor at 24 h. n = 3. **(G)** The CCK-8 method was used to evaluate the effect of the miR-34a mimic on the proliferation of HAoECs at 96 h. n = 3. **(H)** CCK-8 was used to detect the effect of the miR-34a inhibitor on the proliferation of HAoECs at 96 h. n = 3. **(I)** A representative image of SA-β-Gal-stained HAoECs after treatment with the miR-34a mimic or inhibitor at 24 h. n = 5. Bar = 100 μm. **(J)** Statistical analysis of the stained area in **(I)**. n = 5. **(K)** A representative image of γ-H2AX staining of HAoECs after treatment with the miR-34a mimic or inhibitor at 24 h. Bar = 20 μm. **(L)** Statistical analysis of the number of DNA damage foci in **(K)**. n = 5. **(M)** QPCR analysis of the mRNA expression of the inflammatory factors IL-1β, IL-6, IL-8 and TNF-α in HAoECs after miR-34a mimic treatment at 24 h. n = 3. **(N)** QPCR analysis of the mRNA expression of the inflammatory factors IL-1β, IL-6, IL-8 and TNF-α in HAoECs after miR-34a inhibitor treatment at 24 h. n = 3. **(O)** QPCR was used to detect the relative expression levels of the cell cycle-related genes p53, p21, p16 and Rb in HAoECs after miR-34a mimic treatment at 24 h. n = 3. **(P)** QPCR was used to detect the relative expression levels of the cell cycle-related genes p53, p21, p16 and Rb in HAoECs in different treatment groups after miR-34a-5p inhibitor treatment at 24 h. n = 3. **(Q)** Western blotting was used to detect the protein expression of p53, p21, and Rb in HAoECs after miR-34a mimic or inhibitor treatment at 24 h. n = 3. **(R)** Quantitative analysis of the results in **(Q)**. *p < 0.05, **p < 0.01, ***p < 0.001, vs. the NFD group or the control group or mimic-NC; ^##^p < 0.01, ^###^p < 0.001, vs. HFD group or H_2_O_2_ group or inhibitor-NC.

To further explore the mechanism of action of Sodium nitrate in alleviating AS, we searched the GeneCards database for atherosclerosis or aging. The results showed that 276 of the 450 miRNA involved in AS regulation are closely related to aging (Figure 4A). Previous research and retrieval data suggest that AS and aging are inextricably linked (Wang and Bennett, 2012; Björkegren and Lusis, 2022). Therefore, we hypothesize whether sodium nitrate alleviates AS by inhibiting aging. Consequently, we selected miR-34a, which with the highest aging relevance score among the 276 common miRNAs, for following studies, (Figure 4B). To investigate whether miR-34a is involved in the anti-senescent effect of sodium nitrate, we detected the expression levels of miR-34a in the aortic tissue of ApoE^−/−^ mice and HAoECs. The results showed that a 12-week high-fat diet and H_2_O_2_ treatment upregulated the expression of miR-34a, while sodium nitrate treatment downregulated its expression (Figure 4C). We also examined the expression of FGF21, the downstream gene of miR-34a. The results showed that sodium nitrate treatment upregulated the expression of FGF21 both in aortic tissue and in HAoECs (Figure 4D). Based on these results, we speculated that sodium nitrate could regulate the expression of miR-34a and FGF21 and that miR-34a is upstream of FGF21. To verify this hypothesis, a miR-34a-5p mimic and inhibitor were used to treat HAoECs. The results showed that the expression level of FGF21 decreased after miR-34a-5p mimic treatment and increased after miR-34a-5p inhibitor treatment (Figure 4E). The above results suggested that sodium nitrate may alleviate senescence by inhibiting the expression of miR-34a, thereby upregulating the expression of its downstream gene FGF21.

### 3.5 miR-34a could regulate the senescence of HAoECs

To further clarify the role of miR-34a in cellular senescence, miR-34a mimic and inhibitor were used to discover senescence-related phenotypes in HAoECs. The CCK-8 assay results showed that the miR-34a mimic could significantly inhibit the proliferation of HAoECs, and the HAoECs showed almost no proliferative ability after 48 h of treatment (Figure 4G). The miR-34a inhibitor had no effect on the proliferation of HAoECs (Figure 4H). The results also showed that the miR-34a mimic could significantly increase the percentage of SA-β-Gal- and γ-H2AX-positive cells compared to that in the mimic-NC group (Figures 4I–L). Moreover, compared with the inhibitor-NC, the miR-34a inhibitor significantly reduced the percentage of SA-β-Gal- and γ-H2AX-positive cells. The miR-34a mimic upregulated the expression of the inflammatory factors IL-1β, IL-6, IL-8 and TNF-a (Figure 4M) and further exacerbated the inflammatory response. However, the miR-34a inhibitor downregulated the expression of IL-1β, IL-6, IL-8 and TNF-a (Figure 4N). Moreover, the expression levels of the senescence-related genes p53, p21, p16 and Rb were significantly upregulated after treatment with the miR-34a mimic (Figure 4O) and downregulated after treatment with the miR-34a inhibitor (Figure 4P). Changes in the expression of P53, P21 and RB at the protein level were consistent with the above results (Figures 4Q, R). These results indicated that high expression of miR-34a could accelerate cellular senescence in HAoECs, while inhibiting the expression of miR-34a could alleviate cell senescence.

### 3.6 FGF21 is involved in cell senescence

**FIGURE 5:**
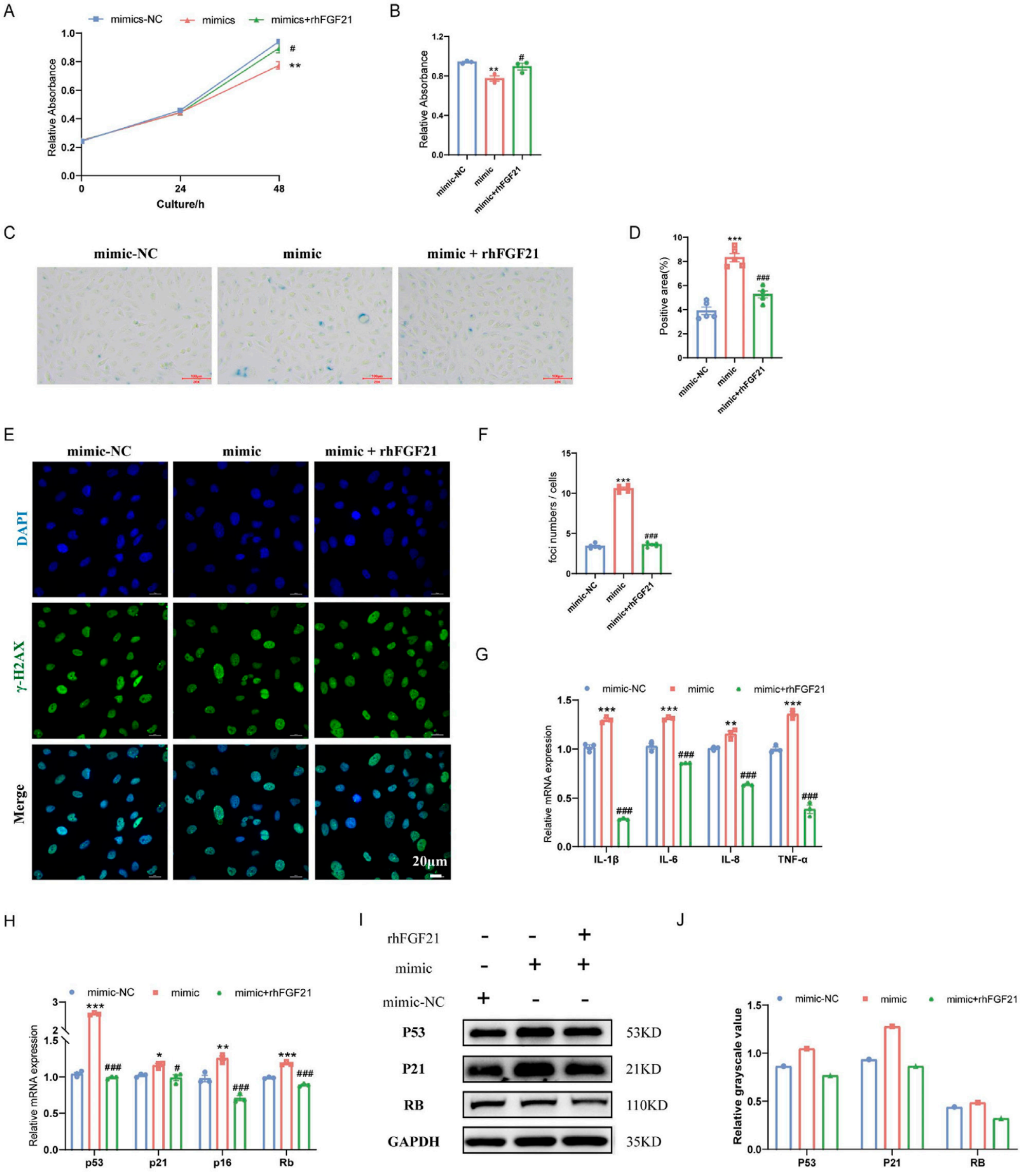
Effect of FGF21 on the senescence of HAoECs. **(A)** A CCK-8 kit was used to detect the effect of rhFGF21 on the proliferation of HAoECs at 48 h. **(B)** Statistical analysis of the HAoEC proliferation ability at 48 h **(A)**. n = 3. **(C)** Representative images of SA-β-Gal-stained HAoECs after treatment with the miRNA-34a mimic or rhFGF21 at 24 h. Bar = 100 μm. **(D)** Statistical analysis of the stained area in **(C)**. n = 5. **(E)** Representative images of γ-H2AX staining of HAoECs after miRNA-34a mimic and rhFGF21 treatment at 24 h. Bar = 20 μm. **(F)** Statistical analysis of the number of DNA damage foci in **(E)**. n = 5. **(G)** QPCR analysis of the mRNA expression of the inflammatory factors IL-1β, IL-6, IL-8 and TNF-α in HAoECs from different treatment groups at 24 h. n = 3. **(H)** QPCR was used to detect the relative mRNA expression of p53, p21, p16 and Rb in HAoECs after treatment with the miRNA-34a mimic or rhFGF21 at 24 h n = 3. **(I)** Western blotting was used to detect the protein expression of P53, P21, and RB in HAoECs after treatment with the miRNA-34a mimic or rhFGF21 at 24 h n = 3. **(J)** Quantitative analysis of the results in **(I)**. *p < 0.05, **p < 0.01, ***p < 0.001, vs. the mimic-NC group; ^#^p < 0.05, ^###^p < 0.001, vs. the mimic group.

To further validate the antiaging effect of FGF21, the recombinant human protein of FGF21 (rhFGF21) was added to treat HAoECs. rhFGF21 reversed the decrease in HAoEC proliferation caused by the miR-34a mimic (Figures 5A, B). In addition, compared with the miR-34a mimic, rhFGF21 treatment reduced the percentage of SA-β-Gal-positive cells (Figures 5C, D) and γ-H2AX-positive cells (Figures 5E, F), decreased the expression of the proinflammatory factors IL-1β, IL-6, IL-8 and TNF-α (Figure 5G), downregulated the mRNA (p53, p21, p16 and Rb) and protein (P53, P21 and RB) expression of the cell cycle-related genes (Figures 5H–J). These results suggested that FGF21 was important in the anti-senescence effect of sodium nitrate.

## 4 Discussion

Cardiovascular disease has become the leading cause of death in humans. Among them, arteriosclerotic cardiovascular disease accounts for the most significant proportion of cases, and the incidence and mortality rate increase annually (Benjamin et al., 2019). The traditional treatment methods for AS mainly include drug treatment, surgical treatment, and lifestyle changes, which have shortcomings, such as limited treatment effects and high risk. In recent years, scholars have found that AS is a class of aging-related diseases that are not only more common in elderly individuals but also accompanied by the senescence of various cells (Tyrrell and Goldstein, 2020). Many treatments for young and middle-aged people have little effect on older people. Therefore, there is an urgent need to develop a new therapy to control this disease, especially when senescence occurs. Elucidating the mechanisms by which senescence promotes atherosclerotic cardiovascular disease will be fundamental to developing new treatments to reduce the burden of atherosclerosis caused by senescence.

Several animals, such as mice, rats, rabbits, and pigs, are often used to establish AS models at present. Among these models, the ApoE^−/−^ mouse has become a standard animal model for AS research because of its simple modeling process and the advantages of vascular lesions being very similar to those of the human body (Emini et al., 2017). In this study, AS model was established by feeding 8-week-old male ApoE^−/−^ mice a high-fat diet for 12 weeks, and each mouse ate about 4 g of diet and drank 5 mL of water per day. During the experiment, mouse body weights were measured at 0, 6 and 12 W. The experimental results showed that the weight of the ApoE^−/−^ mice in the HFD group increased slightly at 6 W and decreased at 12 W. There was no significant change in the body weight of the mice after NaNO3 treatment when compared with HFD group. Lipid-related biochemical indices showed that a high-fat diet could increase the serum levels of CHO and TG. Moreover, it increased the area of aortic Oil Red O-stained plaques and upregulated the expression of TNF-α and other proinflammatory factors in the serum and aorta. This evidence indicated that we successfully established a mouse model of AS, and at the same time, we evaluated the relevant indicators of cell senescence in ApoE^−/−^ mice. The results showed that the percentage of SA-β-Gal-positive cells was increased, and the expression levels of cell cycle-related genes (p53, p21, p16 and Rb) were also increased in the aortas of high-fat diet-fed mice. The above results suggested that ApoE^−/−^ mice exhibit a certain degree of cellular senescence when AS occurs. Nitrates are widely distributed in natural environments, such as food, water, and air. Nitrates used as drugs for cardiovascular diseases can be traced back to ancient China in the 8th century AD (Lundberg et al., 2008). Nitrate effectively alleviates cell senescence and improves cardiovascular function. Therefore, sodium nitrate was selected to intervene in AS and its accompanying cellular senescence in ApoE^−/−^ mice. Lu et al. found that high dietary sodium intake increased systolic blood pressure, but low dietary sodium intake exacerbated atherosclerosis in mice with hypercholesterolemia (Lu et al., 2013). In this study, we selected an intermediate sodium content between high and low concentrations for experimentation. However, we did not study the effects of different nitrate concentrations on aortic atherosclerosis in mice, and we will pay attention to this issue in future experiments. We found that sodium nitrate decreased the concentrations of CHO and TG in the serum of mice, reduced the percentage of positive aortic Oil Red O staining, and decreased the expression levels of inflammatory factors in the serum and aorta in our study. The above results showed that sodium nitrate could alleviate systemic and local inflammation in the aorta and has an anti-AS effect. This observation is not in agreement with the findings of Khambata RS et al. in ApoE^−/−^ mice, where a 12-week nitrate treatment did not alter plaque size in HFD-fed mice (Khambata et al., 2017). This inconsistency may be due to the different components of the high-fat feed used. In Khambata RS’ study, the high-fat feed contained 42 kcal % fat and ∼0.2% cholesterol by weight, while in our study it contained 40 kcal % fat and ∼1.254% cholesterol by weight. There are also studies that are consistent with our experimental results, such as Peng et al.’s research showed that dietary nitrate could reduce the size of atherosclerotic plaques in ApoE^−/−^ mice fed an HFD (Peng et al., 2020). More research is needed to clarify the reasons for the differences between these studies.

Cellular senescence occurs throughout the whole process of AS occurrence and development. DNA damage, cell cycle arrest, massive secretion of inflammatory factors, enhanced SA-β-Gal activity, and mitochondrial dysfunction are the main features of cellular senescence (Lopez-Otin et al., 2023). Based on these findings, researchers have established a number of methods for identifying cellular senescence. However, due to the complexity of cellular senescence regulation, there is currently no way to assess cellular senescence alone. The combination of SA-β-Gal staining, cell cycle arrest, and inflammatory factor expression level is currently a well-recognized method for identifying cellular senescence (Schellenberg et al., 2012). Our experiments showed that sodium nitrate could reduce the percentage of positive aortic SA-β-Gal staining area and downregulate the expression of cell cycle-related genes (p53, p21, p16, and Rb). Therefore, sodium nitrate could alleviate the cellular senescence associated with AS.

H_2_O_2_ is a commonly used inducer that can cause senescence in various cells in a short period of time, simulating the process of oxidative stress experienced by cells during AS occurrence (Wang et al., 2013). Vascular endothelial cells cover the innermost part of the vascular lumen, and structural and functional impairment of these cells could initiate AS (Björkegren and Lusis, 2022). Therefore, HAoECs, a kind of aortic endothelial cell line, were used to establish a cell model induced by H_2_O_2_. Our results showed that H_2_O_2_ could successfully induce cellular senescence in HAoECs. However, after sodium nitrate treatment, cell proliferation was restored, the percentage of SA-β-Gal-positive cells and the number of DNA damage foci decreased, and the expression levels of cell cycle-related genes and inflammatory factors decreased. These results preliminarily validated the results of *in vivo* experiments and suggested that sodium nitrate could alleviate cellular senescence.

miRNAs are noncoding RNA segments with a length of approximately 22 nucleotides that can bind to corresponding mRNAs to regulate gene expression (Ghafouri-Fard et al., 2021). Studies have shown that inhibition of miR-34a expression has therapeutic effects in a variety of aging-related disease models. For example, inhibition of the miR-34a-mediated SIRT1/mTOR signaling pathway attenuates D-gal-induced senescence in rat brain tissue, and alveolar epithelial cell dysfunction is alleviated in aged miR-34a^−/−^ pulmonary fibrosis mice (Kou et al., 2016; Cui et al., 2017). Clinical research data have also shown that the upregulation of miR-34a/b/c expression in peripheral blood mononuclear cells promotes vascular aging and the occurrence of atherosclerotic vascular disease (Gatsiou et al., 2021). Therefore, our experiments explored whether miR-34a can play an important role in the treatment of aortic atherosclerosis through anti-cellular senescence. We found that the expression level of miR-34a was significantly upregulated in ApoE^−/−^ mice fed a high-fat diet, while miR-34a was downregulated considerably after dietary sodium nitrate treatment in our study. To further validate the role of miR-34a in therapeutic effect of NaNO_3_, we first treated HAoECs with a miR-34a mimic and inhibitor. After miR-34a mimic treatment, the percentage of SA-β-Gal-positive cells was increased, cell proliferation was suppressed, the number of DNA damage foci was increased, and the expression levels of cell cycle-related genes and inflammatory factors were upregulated in HAoECs. The effects of the miR-34a inhibitor on HAoECs verified the ability of miR-34a to promote cell senescence. Our results demonstrated the critical role of miR-34a in AS-associated cell senescence.

Studies have shown that miR-34a is involved in regulating the expression of FGF21 (Fu et al., 2014). FGF21 is a hormone-like member of the FGF family, and as a stress hormone, it can be induced by endoplasmic reticulum stress, mitochondrial dysfunction, and autophagy disorders (Salminen et al., 2017). Research has shown that FGF21 can alleviate many age-related metabolic diseases (Youm et al., 2016). To explore whether NaNO_3_ exerts anti-senescence effects by regulating the expression of miR-34a and its downstream gene FGF21, we tested the expression levels of FGF21 in ApoE^−/−^ mice. The results showed that the high-fat diet significantly reduced the expression level of FGF21, and NaNO_3_ treatment upregulated the expression of FGF21, which was consistent with the results at the cellular level.

To further clarify the role of FGF21 in the anti-senescence effect of NaNO_3_, recombinant FGF21 protein was used to treat HAoECs. rhFGF21 rescued the senescence phenotype caused by the miR-34a mimic, as indicated by decreased cell proliferation, increased SA-β-Gal activity, upregulated cell cycle-related genes, and increased inflammatory factor secretion. Thus far, our results suggest that NaNO_3_ might exert anti-senescence effects by regulating the expression of miR-34a and its downstream gene FGF21.

## 5 Conclusion

In conclusion, the results showed that NaNO_3_ could reduce the size of aortic atherosclerotic plaque, inhibit local cell senescence, alleviate local and systemic inflammatory reactions, and downregulate the expression of miR-34a in ApoE^−/−^ mice. Moreover, NaNO_3_ inhibited the expression of miR-34a, upregulated the expression of the downstream gene FGF21, and alleviated H_2_O_2_-induced cellular senescence in HAoECs. This is the first report that NaNO_3_ can reduce cell senescence accompanied by AS through the miR-34a/FGF-21 axis. These findings might lead to the introduction of a new therapy for senescence-related diseases in the future.

## Data Availability

The original contributions presented in the study are included in the article/supplementary material, further inquiries can be directed to the corresponding authors.

## Abbreviations

AS, atherosclerosis; ApoE^−/−^, apolipoprotein E knockout; QPCR, Quantitative Real-time PCR; NOCs, N-nitroso compounds; SA-β-Gal, Senescence-associated β-D-Galactosidase; HAoECs, human arterial endothelial cells; FGF21, fibroblast growth factor 21; CHO, cholesterol; TG, triglyceride; SASP, senescence-related secretory phenotype; DMEM, Dulbecco’s modified Eagle’s medium; FBS, fetal bovine serum; rhFGF21, recombinant human protein of FGF21.
